# Case report: Novel *SCN4A* variant associated with a severe congenital myasthenic syndrome/myopathy phenotype

**DOI:** 10.3389/fped.2022.944784

**Published:** 2022-08-26

**Authors:** Veronika M. Berghold, Mahmoud Koko, Riccardo Berutti, Barbara Plecko

**Affiliations:** ^1^Department of Pediatrics and Adolescent Medicine, Division of General Pediatrics, Medical University of Graz, Graz, Austria; ^2^Department of Neurology and Epileptology, Hertie Institute for Clinical Brain Research, University of Tübingen, Tübingen, Germany; ^3^Institute of Human Genetics, University Hospital Rechts der Isar, Technical University of Munich, Munich, Germany; ^4^Institute of Neurogenomics, Helmholtz Center Munich, German Research Center for Environmental Health, Neuherberg, Germany

**Keywords:** Na_V_1.4 voltage-gated sodium channel, loss of function (LOF), ion channel gene defect, whole exome sequencing, ion channels, sodium channel paralogs, genetic testing, complex I deficiency

## Abstract

We present a now 18-year-old female patient with a severe congenital myopathy phenotype, originally diagnosed as mitochondrial myopathy, however later revealed to constitute a *SCN4A*-related myopathy based on genetic testing. After birth, floppiness, bradycardia and respiratory insufficiency ensued, and moderately reduced mitochondrial complex I activity was found in muscle tissue (tested at 3 weeks and 3 years of age, respectively). She was treated with riboflavin, carnitine, creatine and a ketogenic diet. At the age of 13 years, whole exome sequencing challenged the initial diagnosis by identifying two (compound heterozygous) *SCN4A* variants affecting the highly conserved voltage sensor and pore regions of the voltage-gated sodium channel Na_V_1.4: a known pathogenic loss of function (LOF) variant [c.4360C>T; p.(Arg1454Trp)] and a novel variant of uncertain significance [c.3615C>G; p.(Asn1205Lys)]. For this novel variant, a LOF effect was predicted by *in silico*, clinical and functional evidence from paralog human sodium channels, and the variant was accordingly classified as likely pathogenic. The patient's phenotype is in line with the few published cases of autosomal recessive *SCN4A*-related myopathy. There was limited benefit from treatment with salbutamol and acetazolamide, while pyridostigmine caused side effects at a minor dose. This report highlights the importance of genetic testing in severe myopathies particularly in regard to treatment options and the value of paralog information in evaluating ion channel variations.

## Introduction

*SCN4A* encodes the pore-forming alpha subunit of the voltage-gated sodium channel Na_V_1.4, highly expressed in skeletal muscle. Na_V_1.4 is present in the postsynaptic folds and is responsible for the majority of the cell's inward sodium current, which generates the muscle action potential crucial for skeletal muscle contraction. *SCN4A* belongs to the human *SCNxA* family of alpha subunits which are highly evolutionary conserved with a common structure of four homologous domains (I–IV), each consisting of six transmembrane segments (S1–S6) and a membrane reentrant loop between S5 and S6, showing > 70% amino acid similarity within the gene family (1).

Dominant gain of function *SCN4A* variants cause hyperkalemic or hypokalemic periodic paralysis, paramyotonia congenita and different variants of myotonia (Mendelian Inheritance in Man [MIM] #170500, #613345, #608390, #168300). The much rarer autosomal recessive loss of function (LOF) variants were initially implicated in congenital myasthenic syndrome 16 with response to acetazolamide (CMS16; MIM #614198, Supplementary Table S1) (2–4), while more recent publications delineate a severe, sometimes even lethal myopathic phenotype (Supplementary Table S1) (5, 6).

In this case report we present a now 18-year-old female patient with a severe congenital myasthenic syndrome/myopathy phenotype, initially diagnosed as mitochondrial complex I deficiency, caused by compound heterozygous variants in the *SCN4A* gene and describe a new likely pathogenic *SCN4A* variant.

## Case description

### Clinical presentation

Our patient was born on term to non-consanguineous parents by cesarean section due to breech presentation. The mother reported reduced fetal movements; the pregnancy was otherwise uneventful. Birth weight was 3,250 g (50th percentile), body length 52 cm (65th percentile), head circumference 39 cm (> 99th percentile) and APGAR score of 8 at 1 min, 9 at 5 min and 10 at 10 min. One hour postpartum, she abruptly presented with bradycardia, floppiness and respiratory insufficiency, necessitating intubation and prolonged ventilator dependency resulting in tracheostomy. Enteral feeding was ensured via a gastrostomy tube. Cranial ultrasound was normal. Open muscle biopsy was performed at 3 weeks of age revealing normal findings on light and electron microscopy but decreased complex I activity of the respiratory chain in frozen muscle. Electromyography (EMG) was normal with normal nerve conduction velocity (NCV) and no decrement. At the age of one-year cerebral MRI showed low-grade normal-pressure hydrocephalus.

A second open muscle biopsy, performed at 3 years of age with functional analysis in fresh muscle, confirmed reduced OXPHOS activity of complex I [complex I 14 mU/mg protein (reference range: 28−76), complex I+III 18 mU/mg protein (49–218)]. Blood lactate and creatine kinase (CK) levels were repeatedly normal. Electrolytes in routine check-ups were always in normal range, no hyper- or hypokalemia was detected. She was started on a cocktail of riboflavin (300 mg/d), L-carnitine (50 mg/kg/d), coenzyme Q10 (10 mg/kg/d), creatine (60 mg/kg/d) and a 2:1 ketogenic diet. The need for daily ventilation varied from 8 to 24 h. Intermittent withdrawal of the ketogenic diet led to increased need of ventilatory support.

The patient presents with an elongated hypotonic face, open mouth, convergent strabismus concomitants, bilateral ptosis and external ophthalmoplegia. She never accomplished proper head control or independent sitting. Over the years she had limited improvement in muscle strength, severe general muscle weakness and atrophy. She is unable to lift her head in supine. She uses a wheelchair and can briefly bear weight during transfers. She has satisfying eye-hand-coordination and can lift light objects. Lifting the upper extremity against gravity is possible for approximately 10 seconds, as well as closure of fist, finger spread and flexion of arms with 3/5 muscular strength and rapid fatigue. Deep tendon reflexes are not elicitable.

Cognitive function is preserved with normal speech and good phonation. At the age of 6 years, she entered school with full time assistance and managed to pursue a regular school career.

Due to progressive levoconvex scoliosis, the patient received brace treatment and sitting orthoses, followed by spinal surgery. The patient receives yearly bisphosphonate treatment due to osteoporosis. Regular check-ups did not reveal any further abnormal laboratory findings.

### Genetic evaluation

Seeking to confirm the diagnosis and characterize the underlying genetic defect, whole exome sequencing (WES) was performed at the age of 13 years on the premise of informed consent. Singleton exome (proband only) was performed followed by confirmation of candidate variants and segregation (in the parents) with Sanger sequencing.

#### Whole exome sequencing (WES)

WES and primary data analysis were performed at the Helmholtz Center Munich and at the Technical University of Munich (Munich, Germany). We used SureSelect Human All Exon v6 kit (60Mb) to capture exonic regions and sequencing was performed on an Illumina HiSeq4000 platform.

An in-house bioinformatics pipeline was used for sequence alignment and variant annotation and filtration as previously described (7, 8). In summary, read alignment was performed with BWA and variant calling (for short and copy number variants) with samtools, the Genome Analysis Toolkit (GATK), Pindel, ExomeDepth. The GATK-based variant calling adhered to the GATK Best Practices (9). Variant annotation was performed with in-house scripts incorporating dbSNP, gnomAD, ClinVar and OMIM data.

**Figure 1:**
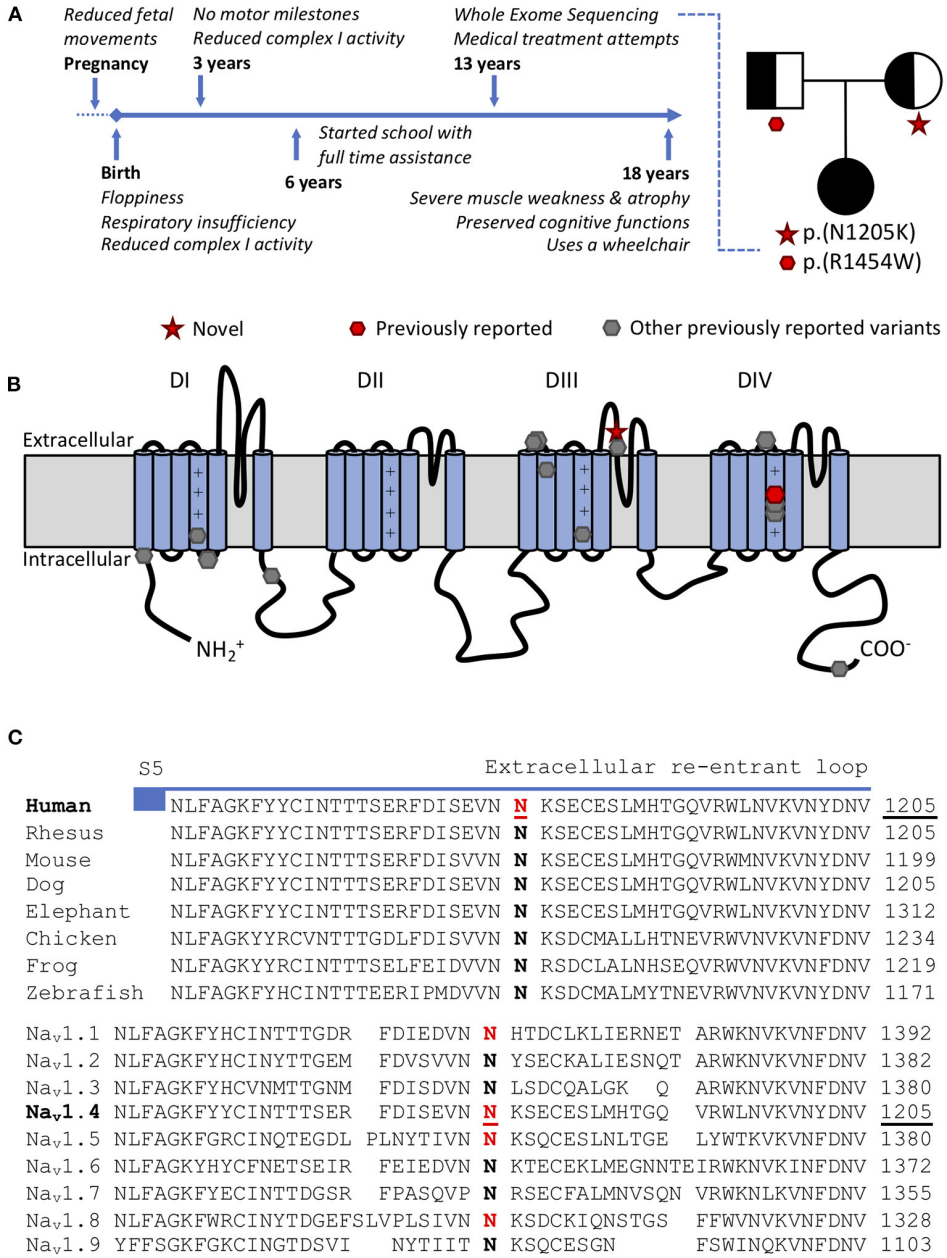
**(A)** Time-line and family tree. Each parent is asymptomatic and a carrier for one variant. **(B)** Scheme of the pore forming alpha subunit of Na_V_1.4 with locations of CMS/myopathy-linked *SCN4A* variants: red p.(N1205K) and p.(R1454W) (compound heterozygous), gray (other previously reported variants): p.R104H, p.R225W, p.S246L, p.Q470X, p.A1049VfsX50, p.D1059N, p.R1059X, p.R1135C, p.C1205F, p.V1442Q, p.R1457H p.R1460W/Q, p.H1782QfsX85. **(C)** Amino acid conservation in the extracellular S5-S6 reentrant loop region of domain III among Na_V_1.4 homologs. The variant *SCN4A*:p.N1205K affects a highly conserved amino acid in Na_V_1.4 both in orthologs (top) and human paralogs (bottom). Variants paralogous to p.(N1205K) (marked in red) in Na_V_1.1 (*SCN1A*:p.(N1392K), twice), Na_V_1.5 (*SCN5A*:p.(N1380K), thrice) and Na_V_1.8 (*SCN10A*:p.(N1328K), once) were reported in patients with neurological or cardiac phenotypes and electrophysiological evaluation in Na_V_1.5 supported a loss-of-function effect.

The results of WES challenged the diagnosis of mitochondrial myopathy. The patient was found to carry two variants in the *SCN4A* gene [NM_000334.4: c.3615C>G, p.(Asn1205Lys) and c.4360C>T, p.(Arg1454Trp)]. Her parents were asymptomatic, each confirmed by segregation analysis to carry one allele (Figure 1A). Next, we evaluated the evidence for pathogenicity and then classified these variants based on the American College of Medical Genetics and Genomics/Association for Molecular Pathology (ACMG/AMP) criteria (10).

#### Evidence of variant pathogenicity from studies of human *SCN4A* and *SCNxA* paralogs

*SCN4A:*p.(R1454W), located in the voltage sensor of domain IV of Na_V_1.4, has been previously reported as disease-causing in the homozygous state, with functional evidence showing LOF (4). *SCN4A*:p.(N1205K) has neither been described in patients nor functionally characterized before. It is located in the extracellular part of S5–6 reentrant loop of domain III of Na_V_1.4 (Figure 1B). This region forming the channel pore is highly conserved among multiple species and within the human *SCNxA* family (Figure 1C). Mutations in this region are likely to impair channel function (11).

The human *SCNxA* family of alpha subunits of the voltage gated sodium channels consists of nine family members, with several disease associations. To predict the functional effect of this new variant *SCN4A*:p.(N1205K), we utilized multiple approaches based on paralog conservation (Figure 1C). First, we used funNCion (http://funNCion.broadinstitute.org) a recently developed *in silico* tool predicting functional effects in voltage-gated sodium and calcium channels (12). We obtained a probability of pathogenicity of 0.88 and probability of 0.79 of a LOF effect. Second, we evaluated the phenotypes associated with paralogous variants (i.e., variants that affect an equivalent position and cause the same amino acid change in another Na_V_1.x channel) and last, we searched for functional evidence from the *SCNxA* family of genes.

We found that paralogous variants of *SCN4A*:p.(N1205K) have been described in three other *SCNxA* family members. p.(N1392K) in *SCN1A* (encoding Na_V_1.1, mainly expressed in central neurons) was described in two patients. Through next generation sequencing, *SCN1A:*p.(N1392K) was found in a 7 month old boy with unknown type of early developmental and epileptic encephalopathy (DEE) (13). Further, *SCN1A:*p.(N1392K) was confirmed in a male with febrile seizures (FS) in a study with proband-only medical exome sequencing (14). *SCN1A* LOF variants underlie a wide spectrum of epilepsies from Generalized Epilepsy Febrile Seizures Plus (GEFS+) to Dravet syndrome.

p.(N1380K) in *SCN5A* (encoding Na_V_1.5, expressed in cardiac muscles) was reported in three patients with cardiac disease along the spectrum of Brugada syndrome, a condition that is linked to *SCN5A* LOF variants, increasing the risk for sudden cardiac death (15–17). Another paralogous variant p.(N1328K) in *SCN10A* (encoding Na_V_1.8, expressed in the dorsal root ganglion) was reported in a patient who showed signs of Brugada syndrome after treatment for paroxysmal atrial fibrillation (18). Whether the variant directly affects Na_V_1.8 (which is also expressed in cardiac myocytes) or affects the function of Na_V_1.5 indirectly is not clear, the authors argued, and would require more studies (18). To our knowledge there are no other paralogous variants reported in the literature.

Electrophysiological evaluation of p.N1380K in Na_V_1.5 with high-throughput automated patch clamping confirmed LOF with minimal residual sodium current (19). According to these data, the variant in *SCN5A*:p.(N1380K) has been reclassified by the authors from “variant of uncertain significance” to “likely pathogenic” according to the ACMG/AMP classification scheme (10, 19).

#### Classification of *SCN4A* variants according to the ACMG/AMP criteria

The ACMG/AMP classification (10) was applied using InterVar tool (20); the already known variant *SCN4A:*p.(R1454W) was classified as “likely pathogenic” (PS3, PM1, PM2, PP3) and the newly described variant *SCN4A:*p.(N1205K) has been initially considered a “variant of uncertain significance” (PM2, PM3, PP3). Using the above described evidence of pathogenicity from paralogs, the classification of *SCN4A*:p.(N1205K) has been updated to “likely pathogenic” with two additional supporting criteria (PS1 & PS3 as supporting). The details of the pathogenicity evidence and criteria are given in Table 1.

**Table 1:** Classification of two compound heterozygous *SCN4A* variants according to the ACMG/AMP criteria.

**Variant**	**Criteria**		**Strength of pathogenicity evidence**	**Classification and ClinVar accession**
*SCN4A:*p.(R1454W)	PS3	Well-established *in vitro* functional studies showed loss-of-function effect on Na_V_1.4 (enhanced inactivation and slow recovery)	Strong (1x)	Likely pathogenic SCV002549165
	PM1	Located in S4 which is a mutational hotspot and a critical and well-established functional (voltage sensor) domain	Moderate (2x)	
	PM2	Extremely low frequency in gnomAD r2.1 (MAF 0.00001609 and no homozygotes)		
	PP3	Multiple lines of computational evidence support a deleterious effect (REVEL: 0.82, CADD: 25.9, GERP++: 2.7, PPh2: 0.99, SIFT: 0)	Supporting (1x)	
*SCN4A:*p.(N1205K)	PM2	Absent from controls (gnomAD r2.1)	Moderate (2x)	Likely pathogenic SCV002549166
	PM3	Detected in *trans* with a likely pathogenic variant in a gene associated with a recessive disease		
	PP3	Multiple lines of computational evidence support a deleterious effect (REVEL: 0.83, CADD: 28.7, GERP++: 3.8, PPh2: 0.97, SIFT: 0).	Supporting (3x)	
	PS1	Same amino acid change as a previously established pathogenic variant in two paralog channels (*SCN1A, SCN5A*)		
	PS3	Well-established *in vitro* functional study showed a loss-of-function effect in the paralog channel Na_V_1.5 (almost no sodium current)		

### Outcomes and follow-up

Therapeutic attempts following this genetic diagnosis were of limited benefit. Pyridostigmine administered at a low therapeutic dose (8 mg/kg/d) caused immediate side effects such as nausea, diarrhea, hypersalivation and inappetence while only a small increase in muscular strength could be observed. An attempt to increase the dose up to 16 mg/kg/d caused an aggravation of side effects and pyridostigmine was eventually discontinued. Salbutamol administered at maximum dose (0.3 mg/kg/d) showed a short term and moderate increase in muscular strength. Treatment with acetazolamide monotherapy (17 mg/kg/d) led to some improvement in ventilatory support, facial expression, range of eye movements and ptosis. Besides mild polyuria, no other side effects were reported. The current combination therapy of acetazolamide (17 mg/kg/d) and salbutamol (0.2 mg/kg/d) led to minor clinical improvement, restricted to endurance of muscle activity (Supplementary Table S1).

Currently, at the age of 18 years the patient is able to stand with support during transfer and to lift her stretched arms against gravity for approximately 10 seconds with increasing tremor, as well as her legs for approximately 7 seconds. She has neck, axial and limb weakness, rapid muscular fatigue and generalized muscle atrophy, and continues to use a wheelchair. Current weight is 30 kg (far below 3rd percentile), current length is 147 cm (below 3rd percentile).

#### Patients perspective

The patient and her parents were content to receive a genetic diagnosis. However, the treatment with pyridostigmine showed immediate side effects, which were perceived very uncomfortable. She appreciates some limited benefit from her actual treatment with salbutamol. Besides she is a very friendly, open-minded young woman, who seems to accept her diagnosis and tries to live her life as independently as possible, with the support of her parents. She managed to finish commercial school and attempts to find a sheltered job as a clerk.

## Discussion

*SCN4A* pathogenic LOF variants are extremely rare. Initially linked to CMS16 (2–4), a severe, sometimes even lethal myopathic phenotype has been described more recently (5, 6). The phenotype associated with these autosomal recessive variants is characterized by severe muscular insufficiency most often starting at birth, sometimes with clinical improvement over time (Supplementary Table S1). This phenotypic spectrum is possibly related to the degree of functional impairment of the SCN4A protein (5, 6). Complete LOF in both alleles is likely not compatible with life and was associated with still birth and congenital anomalies (6).

We report here a patient with a severe autosomal recessive CMS/myopathy phenotype of neonatal onset, implicating compound heterozygosity for the LOF variant *SCN4A:*p.(R1454W) and a novel variant *SCN4A:*p.(N1205K) in *SCN4A*-related disease. The previously reported patient with homozygous *SCN4A:*p.(R1454W) alleles suffered from permanent but fluctuating muscle weakness with acute and reversible attacks up to full paralysis, however was able to walk (4), in contrast to our patient, emphasizing the potential impact of the newly identified variant *SCN4A:*p.(N1205K) in causing a more severe phenotype.

Deciphering the pathogenicity of new ion channel variants is not always straight forward in the absence of functional data. True disease-causing alleles in *SCNxA* genes may be difficult to set apart from incidental findings. For instance, the significance of the paralogous variant *SCN10A:*p.(N1328K) is uncertain since the association between *SCN10A* and Brugada syndrome is not well established. Moreover, complex presentations may be due to multiple co-existing variants in several genes. For example, one of the patients with the paralogous variant *SCN5A*:p.(N1380K) diagnosed with Brugada syndrome additionally suffered of myotonic dystrophy type 2 (genetically confirmed) with symptoms of abnormal fatigue and hand myotonia (15).

Whereas the pathogenicity of *SCN4A:*p.(R1454W) was functionally validated, *SCN4A:*p.(N1205K) was initially considered of uncertain significance. We therefore leveraged the conservation of the mutated amino acid across the human *SCNxA* family of genes to clarify the effects of *SCN4A:*p.(N1205K).

All nine *SCNxA* family members have a mutual evolutionary descent and a highly conserved amino acid sequence structure, allowing fairly accurate predictions of functional effects. Paralog-based *in silico* predictions suggested a LOF effect with high probability (12). Additionally, a recent systematic analysis of functionally assessed variants in the nine voltage-gated sodium channel genes (11) showed that variants across all four S5–6 reentrant loop regions were predominantly (in 91% of cases) associated with LOF. Furthermore, regardless of which one of the *SCNxA* family member was impaired, paralogous variants in this region showed similar functional consequences in 92% of cases (11). The authors highlight that biophysical characterization of variants in one *SCNxA* gene family member is useful to estimate channel function in other *SCNxA* gene family members when experimental data is unavailable (11).

In addition to its location in the S5-6 reentrant pore loop, the phenotypes of individuals with paralogous variants in *SCN1A* (DEE, FS) and *SCN5A* (Brugada syndrome) and the functional data from *SCN5A* (markedly reduced current density) indicate that *SCN4A:*p.(N1205K) is a likely LOF variant. Although all of the above-described paralogous variants are reported in heterozygous state, this is consistent with the modes of inheritance of their respective disorders. For recessive presentations caused by Na_V_1.4 LOF, haploinsufficiency is tolerated as it preserves some function and both parents of our proband were healthy. In contrast, for Na_V_1.5 in the heart and Na_V_1.1 in the brain, haploinsufficiency is not well tolerated (21).

Recurrence and functional evidence of altered protein function are strong pathogenicity criteria (PS1 & PS3) according to the ACMG/AMP framework, albeit when established in the same gene. As discussed above, the concordance in functional effects among paralogous variants exceeds 90%. We therefore incorporated these data in our classification in a conservative manner; the criteria PS1 & PS3 were downgraded from strong to supporting evidence of pathogenicity with respect to lack of data from the same gene, which we consider an important limitation of this report.

Patients with CMS/myopathy syndrome due to variants in *SCNA4* show poor response to pyridostigmine but may benefit from treatment with acetazolamide (Supplementary Table S1). This was also observed in our patient. Salbutamol as a therapeutic attempt should be considered in these patients as well, since the patient reported by Elia et al. (5) and our patient showed some benefit in muscular strength. Reduced complex I deficiency seen in our patient most likely represents a secondary phenomenon as has been described with this disease (6) and other neuromuscular disorders (22, 23).

Our patient was born at a time of restricted possibilities regarding genetic testing and was considered to suffer from mitochondrial myopathy, whereas WES performed more than 10 years later revealed the correct diagnosis of a *SCN4A*-related myopathy caused by LOF Na_V_1.4 variants, which facilitated informed counseling about the cause of the disease and offered insights into new treatment options with some (although limited) benefit. We therefore highlight the importance of genetic testing in patients with neuromuscular disorders in all age groups in order to provide better clinical care and consequently improve the quality of life for patients.

## Data availability statement

The original contributions presented in the study are included in the article, further inquiries can be directed to the corresponding author/s.

## Ethics statement

This case report was approved by the Institutional Ethics Committee of the Medical University of Graz, Austria (33-573 ex 20/21). Informed consent to carry out the genetic investigations was signed by the patient's parents. A written informed consent for publication of this report was obtained from the patient described in this case report as well as her mother.

## Author contributions

BP conceived the presented idea and collected the patient's data. VB and BP conceptualized and designed the case report. VB drafted the initial manuscript and contributed to the interpretation of results. MK and RB performed the analysis and interpretation of data. All authors critically reviewed and revised the manuscript, approved the final manuscript as submitted, and agree to be accountable for all aspects of the work.

## Funding

VB and BP are supported by the Medical University of Graz. MK was supported by the German Academic Exchange Office (DAAD personal funding program number 57214224). This work was supported by the German Federal Ministry of Education and Research, network on rare neurological ion channel disorders 'Treat-ION' (01GM1907A) and the foundation 'no epilep' to H. Lerche.

## Conflict of interest

The authors declare that the research was conducted in the absence of any commercial or financial relationships that could be construed as a potential conflict of interest.

## Publisher's note

All claims expressed in this article are solely those of the authors and do not necessarily represent those of their affiliated organizations, or those of the publisher, the editors and the reviewers. Any product that may be evaluated in this article, or claim that may be made by its manufacturer, is not guaranteed or endorsed by the publisher.
