# Supplementary material for: Case report: Novel SCN4A variant associated with a severe congenital myasthenic syndrome/myopathy phenotype
Source: Front Pediatr. 2022 Aug 26;10:944784. doi: 10.3389/fped.2022.944784 (PMC9462513; doi:10.3389/fped.2022.944784)
Supplement: Supplementary file 1 [file Table_1.pdf]

Table S1: Congenital myasthenic syndrome/myopathy due to *SCN4A* LOF variants. This supplementary table summarizes the so far reported individuals with *SCN4A*-related myopathy due to loss of function variants, their presentations, and treatment attempts.

***SCN4A* Variants**	**Patient and Age at onset**	**Clinical features**	**Treatment attempts**	**References**
Compound heterozygous:<br><br>**p.N1205K**<br>(not characterized but predicted to profoundly reduce current amplitude)<br><br>**p.R1454W**<br>(affects gating, enhances fast and slow inactivation, slows recovery from inactivation, modest slow current decay)	18-year-old female<br><br>onset: since birth	congenital hypotonia, respiratory insufficiency and mechanical ventilation, PEG tube, hypotonic face, elongated face, bilateral ptosis, ophthalmoplegia, normal speech, neck, axial and limb weakness, delayed motor development (no milestones achieved), uses a wheel-chair, not able to lift head, able to support weight during transfer, able to lift arms against gravity for ten seconds with increasing tremor and legs for seven seconds, muscular strength 3/5 and rapid fatigue, generalized muscle atrophy, scoliosis<br><br>no decrement in nerve stimulation	pyridostigmine (8mg/kg/d), small increase in muscular strength<br>low therapeutic dose immediate side effects (nausea, diarrhea, hypersalivation, inappetence), dose increase (16mg/kg/d) aggravation of side effects, discontinued<br><br>acetazolamide (17mg/kg/d), improvement in facial expression, range of eye movements and ptosis, reduced ventilatory support, little side effect (mild polyuria)<br><br>combination therapy: acetazolamide (17mg/kg/d) and salbutamol (0.2mg/kg/d) overall limited clinical improvement (especially endurance of muscle strength), satisfactory respiratory situation (4-8 hours off ventilation/day), no severe side effects	This study<br>((1) for functional studies on R1454W)
Compound heterozygous:<br><br>**p.S246L**<br>(affects gating, mixed effects, left shift in voltage dependence of fast activation, incomplete & left shift in slow inactivation)<br><br>**p.V1442Q**<br>(affects gating, prominent left shift in the voltage dependence of fast inactivation)	20-year-old female<br><br>onset: since birth	facial, ocular, truncal and limb muscle fatigue, worsens by activity, unable to walk less than 100 meters, elevate arm for max. 20 seconds, recurrent attacks of respiratory and bulbar paralysis (3-30 minutes, 1-3 times a month), ventilator support during apnea attacks, ptosis, delayed motor development, mentally retarded<br><br>decrement in nerve stimulation	pyridostigmine (3x 60-120mg/d) improved endurance<br><br>combination therapy with acetazolamide (2x 250mg/d) prevented attacks of respiratory and bulbar weakness	(2)

Homozygous:<br><br>**p.R1457H**<br>(affects gating, slow recovery)	57-year-old female<br><br>onset: unknown, remembers spells from age 12 years	longstanding muscle fatigue, ptosis, ophthalmoplegia, weakness in neck flexion and extension and knee flexion, attacks of generalized muscle weakness for approx. 1 hour (more intense over time), unable to do regular housework, slow gait, occasional need of a walker<br><br>decrement in nerve stimulation	pyridostigmine ineffective<br><br>acetazolamide not administered due to kidney stones	(3)
Homozygous:<br><br>**p.R1454W**<br>(affects gating, enhances fast and slow inactivation, slows recovery from inactivation, modest slow current decay)	26-year-old female<br><br>onset: since birth	congenital hypotonia, permanent but fluctuating muscle weakness (attacks up to full paralysis, 2-3 times a week), respiratory and swallowing difficulties during attacks, fluctuating ptosis, hypomimia, ophthalmoplegia, muscle weakness in limb girdles, proximal muscles, neck and trunk flexion, delayed motor development, waddling gait, unable to run<br><br>no decrement in nerve stimulation	pyridostigmine (up to 480mg/d) partially beneficial, no benefit on attacks<br><br>acetazolamide (250mg/d) with potassium (up to 4,500mg/d) no clinical improvement<br><br>all treatments but potassium was discontinued, better life quality	(1)
Homozygous:<br><br>**p.R1460W**<br>(markedly reduced current amplitude, affects gating with mixed effects, left shift in voltage dependence of fast inactivation, slow current decay, slow entry in & enhanced recovery from fast inactivation)	Female (unknown age)<br><br>onset: since birth	congenital hypotonia, respiratory insufficiency, mechanical ventilation and PEG tube at infancy, weakness in axial and limb muscles, delayed motor development, able to walk, day-to-day variability in strength and motor performance<br><br>no decrement in nerve stimulation	salbutamol inhaled improvement of strength and breathing, not observed with oral therapy<br><br>pyridostigmine no improvement of strength<br><br>acetazolamide improvement of mobility	(4)
Compound heterozygous:<br><br>**p.R1460Q**<br>(markedly reduced current amplitude, affects gating with mixed effects, left shift in voltage dependence of fast inactivation, slow current decay, slow entry in & enhanced recovery from fast inactivation)<br><br>**p.R1059X**	30-year-old female<br><br>onset: since birth	congenital hypotonia, respiratory and feeding difficulties (improvement over time), mild dysphonia, ptosis, larynx spasms, muscle weakness and fatigue, severe weakness in neck and facial muscles, muscle strength 4/5, delayed motor development, able to walk, fatigability when climbing stairs<br><br>decrement in nerve stimulation	no information provided	(4)

(predicted complete loss-of-function via non-sense mediated decay)<br><br>Additionally, CLCN1 mutation p.R894X				
Compound heterozygous:<br><br>**p.R104H**<br>(no detectable sodium currents)<br><br>**p.R1135C**<br>(affects gating, mixed effects, enhances fast inactivation, causes gating pore current)	14-year-old female<br><br>onset: since birth	congenital hypotonia, difficulties with sucking during infancy, elongated face, mild ophthalmoplegia, mild facial, neck flexion, axial and limb weakness, delayed motor development, slow waddling gait, wheelchair for longer distances, ongoing improvement in strength and motor skills over time, generalized muscle atrophy, scoliosis<br><br>no decrement in nerve stimulation	improvement in strength with regular oral salbutamol	(5)
Compound heterozygous:<br><br>**p.R225W**<br>(reduced current amplitude, right shift in voltage dependence of fast activation<br><br>**p.C1209F**<br>(no detectable sodium currents)	35-year-old female<br><br>onset: since birth	congenital hypotonia, non-invasive respiratory support, elongated face, ophthalmoplegia, mild facial, neck flexion, axial and limb weakness, delayed motor development, slow waddling gait, ongoing improvement in strength and motor skills over time, deterioration of motor skills from 30 years, generalized muscle atrophy<br><br>decrement in nerve stimulation	no information provided	(5)
Compound heterozygous:<br><br>**p.Q470X**<br>(predicted complete loss-of-function via non-sense mediated decay)<br><br>**p.H1782QfsX65**<br>(nine amino acids longer than the wild-type with	2.5-year-old male<br><br>onset: since birth	congenital hypotonia, feeding and respiratory difficulties, NG/PEG tube, facial, neck, axial and limb weakness, delayed motor development, not yet walking, uses wheelchair, improvement in strength and motor skills over time, language and fine motor delay, generalized muscle atrophy<br><br>no decrement in nerve stimulation	no information provided	(5)

altered C-terminal sequence, no change detected upon heterologous expression)				
Compound heterozygous:<br><br>**p.D1069N**<br>(subtle reduction in current amplitude, right shift in voltage dependence of fast activation, right shift in voltage dependence of fast inactivation)<br><br>**p.A1049VfsX50**<br>(predicted complete loss-of-function via non-sense mediated decay)	8-year-old female<br><br>onset: since birth	congenital hypotonia, respiratory insufficiency and mechanical ventilation until 2 years, NG/PEG tube until 4 years, elongated face, mild facial, neck, axial and limb weakness, delayed motor development, improvement in strength and motor skills over time, uncoordinated gait, persistent oromotor weakness, language and fine motor delay, generalized muscle atrophy, scoliosis<br><br>no decrement in nerve stimulation	no information provided	(5)

References:

(1) Habbout K, Poulin H, Rivier F, Giuliano S, Sternberg D, Fontaine B, et al. A recessive Nav1.4 mutation underlies congenital myasthenic syndrome with periodic paralysis. *Neurology* (2016) **86**:161-169. doi: 10.1212/WNL.0000000000002264 [doi].

(2) Tsujino A, Maertens C, Ohno K, Shen XM, Fukuda T, Harper CM, et al. Myasthenic syndrome caused by mutation of the SCN4A sodium channel. *Proc Natl Acad Sci U S A* (2003) **100**:7377-7382. doi: 10.1073/pnas.1230273100 [doi].

(3) Arnold WD, Feldman DH, Ramirez S, He L, Kassar D, Quick A, et al. Defective fast inactivation recovery of Nav 1.4 in congenital myasthenic syndrome. *Ann Neurol* (2015) **77**:840-850. doi: 10.1002/ana.24389 [doi].

(4) Elia N, Nault T, McMillan HJ, Graham GE, Huang L, Cannon SC. Myotonic Myopathy With Secondary Joint and Skeletal Anomalies From the c.2386C>G, p.L769V Mutation in SCN4A. *Front Neurol* (2020) **11**:77. doi: 10.3389/fneur.2020.00077 [doi].

(5) Zaharieva IT, Thor MG, Oates EC, van Karnebeek C, Hendson G, Blom E, et al. Loss-of-function mutations in SCN4A cause severe foetal hypokinesia or 'classical' congenital myopathy. *Brain* (2016) **139**:674-691. doi: 10.1093/brain/awv352 [doi].
